# Therapeutic Effects of *Zymomonas mobilis* on Experimental DSS-Induced Colitis Mouse Model

**DOI:** 10.3390/microorganisms11112793

**Published:** 2023-11-17

**Authors:** Manuela Maragno do Almo, Isabel Garcia Sousa, Vitor Guimarães Olinto, Sylvia Barbosa Pinhate, José Luiz de Paula Rôlo Jivago, Davi Emanuel Ribeiro de Sousa, Márcio Botelho de Castro, Marciano Régis Rubini, Andrea Queiroz Maranhão, Marcelo Macedo Brigido

**Affiliations:** 1 Department of Cell Biology, Institute of Biological Sciences, University of Brasilia, Brasilia 70910-900, Brazil; manu.maragno5@gmail.com (M.M.d.A.); isagarsousa@gmail.com (I.G.S.); olintogvitor@gmail.com (V.G.O.); sylviapinhate@gmail.com (S.B.P.); andreaqm@unb.br (A.Q.M.); 2 Molecular Pathology Graduation Program, Medicine Faculty, University of Brasilia, Brasilia 70910-900, Brazil; 3 Molecular Biology Graduation Program, Institute of Biological Sciences, University of Brasilia, Brasilia 70910-900, Brazil; 4 Institute of Biological Sciences, University of Brasilia, Brasilia 70910-900, Brazil; jivago@unb.br; 5 Veterinary Pathology Laboratory, Campus Darcy Ribeiro, University of Brasilia, Brasilia 70910-900, Brazil; daviers@hotmail.com (D.E.R.d.S.); mbcastro@unb.br (M.B.d.C.); 6 Laboratory of Genetics and Molecular Biology, Embrapa Agroenergy, Brasilia 70770-901, Brazil; mrrubini@gmail.com; 7 Institute for Immunology Investigation, National Institute of Science and Technology (iii-INCT), Brasilia 70067-900, Brazil

**Keywords:** *Zymomonas mobilis*, DSS-induced colitis, microbiota, immunomodulation, probiotics

## Abstract

*Zymomonas mobilis*, a Gram-negative bacteria observed in some popular beverages, is considered safe and has been studied for its potential therapeutic benefits. In this study, we explored its effects on the inflammatory process, tissue integrity, differential gene expression, and microbiota composition in an experimental dextran sulfate sodium (DSS)-induced colitis model in mice. As a result, *Z. mobilis* alleviated the symptoms caused by DSS administration, as indicated by reduced weight loss, disease activity index, a significant reduction in the colon weight/length ratio, and histopathological improvement. Also, *Z. mobilis* could restore the mucosal barrier as well as increase the expression of *Muc3* and *Ocln* genes. An analysis of 16S rRNA sequences showed that *Z. mobilis* alters gut microbiota, increasing *Akkermansia muciniphila* abundance and decreasing *Escherichia coli*. Furthermore, *Z. mobilis* seems to be involved in potentiating a regulatory phenotype by inducing immunomodulatory genes like *Tgfb*, *Il5*, *Il10*, and *Foxp3* and reducing the relative mRNA expression of proinflammatory cytokines *TNF*, *Il6*, and *Il17*. Our data suggest that *Z. mobilis* could alleviate disease progression and be considered a possible probiotic adjuvant for pathologies of the bowel.

## 1. Introduction

The Gram-negative bacteria *Zymomonas mobilis* was initially identified in the ritualistic fermented beverage pulque, popular in Mesoamerica. However, it attracted attention for its anaerobic metabolism, which uses the alternative Entner–Doudoroff (ED) pathway, and its efficient ethanol production. It is also widely studied for its metabolite production of ethanol, sorbitol, succinic acid, levan, and lactate [1,2].

The use of *Z. mobilis* as a probiotic precedes its technological interest. Liquid cultures of *Z. mobilis* were used to treat patients with intestinal disorders, such as chronic colitis [3]. Its probiotic potential and improvement of immune function were also evaluated in rats [4]. In humans, *Z. mobilis* broth effectively regulated intestinal transit [5]. This bacterium has a differentiated physiology which is beginning to be understood, giving it unique nuances that can be better explored for its therapeutic use. Also, *Z. mobilis* is generally recognized as safe (GRAS status), which ensures its use in products for human consumption [6].

Ulcerative colitis (UC) and Crohn’s disease belong to the intestinal bowel disease (IBD) group, characterized as idiopathic disorders that cause inflammation in the gastrointestinal tract (GIT). Currently, UC has no cure and treatments are aimed at controlling and reducing the disease’s symptoms. Treatment possibilities include the use of corticosteroids, anti-inflammatories such as aminosalicylates, immunosuppressants, as well as the administration of immunotherapies [7,8].

Probiotic microorganisms appear as an adjuvant for IBD. In UC, they can help restore the epithelial/mucosal barrier, increasing the thickness of the mucus and the proteins that make up the intercellular junctions [9]. Also, they can increase the anti-inflammatory immune response by upregulating immunoglobulins, defensins, and bacteriocins in the lumen [10,11]. Several species of bacteria (e.g., *Lactobacillus* and *Bifidobacterium*) and fungi (e.g., *Saccharomyces boulardii*) have been evaluated for their beneficial effects on humans [12].

To test whether UC patients could benefit from consuming *Z. mobilis* as a probiotic, we tested it in a mouse model of colitis. Experimental colitis was induced with dextran sulfate sodium (DSS) in mice receiving *Z. mobilis*. The course of colitis was evaluated, and its effect on the inflammatory process was tested by analyzing its role in gene differential expression, in its impact on the microbiota of these animals, and in tissue recomposition. We found that treating mice orally with *Z. mobilis* ameliorates disease indexes and immunological and molecular markers. Moreover, *Z. mobilis* treatment partially reversed the alterations in the gut microbiota in colitis mice, mainly by increasing the abundance of potentially beneficial bacteria and decreasing the abundance of potentially harmful bacteria. This is the first report on *Z. mobilis* use in the experimental model of ulcerative colitis.

## 2. Materials and Methods

### 2.1. Bacterial Strain and Growth Conditions

*Zymomonas mobilis* (ZM4—ATCC 31821) cultures were grown in RM medium (2% *w*/*v* glycose; 1% *w*/*v* yeast extract; 0.1% *w*/*v* NH_4_SO_4_; 0.1% *w*/*v* MgSO_4_; 0.2% *w*/*v* KH_2_PO_4_; pH 6) at 30 °C and 200 rpm overnight. Doses were made with *Z. mobilis* at a concentration of 5 × 10^8^–5 × 10^9^ CFU/mL (Colony Forming Units per mL) with glycerol 25% (*v*/*v*) and stored at −80 °C freezer. Before oral gavage, the doses were centrifuged (5000× *g* at 4 °C for 12 min) and resuspended in 100 µL of sterile saline.

### 2.2. Experimental Design

All animal procedures followed the rules of the Ethical Principles in Animal Experimentation adopted by the Ethics Committee on Animal Experimentation (CEUA/ICB-UnB/Brazil) and approved by CEUA with protocol number 78/2018. Female C57BL/6 mice (10 weeks) were purchased from the CEMIB (Centro Multidisciplinar para Investigação Biológica) of Universidade Estadual de Campinas (Unicamp—Campinas, Brazil). Animals from the same experimental group were housed in a single cage in a controlled temperature (25 °C) room with a 12 h light/dark cycle and ad libitum access to food and water.

The experiment consisted of 24 mice randomly distributed in 4 groups, and it lasted 10 days. The groups were (i) healthy negative control group (NC); (ii) colitis-positive control group (DSS); (iii) experimental group (ZM); and (iv) experimental colitis-positive plus *Z. mobilis* group (DZM). The ZM and DZM groups were administered with 100 µL of *Z. mobilis* dose once daily by oral gavage while the NC and DSS groups were administered with 100 µL of sterile saline.

### 2.3. DSS-Induced Colitis

**Figure 1:**
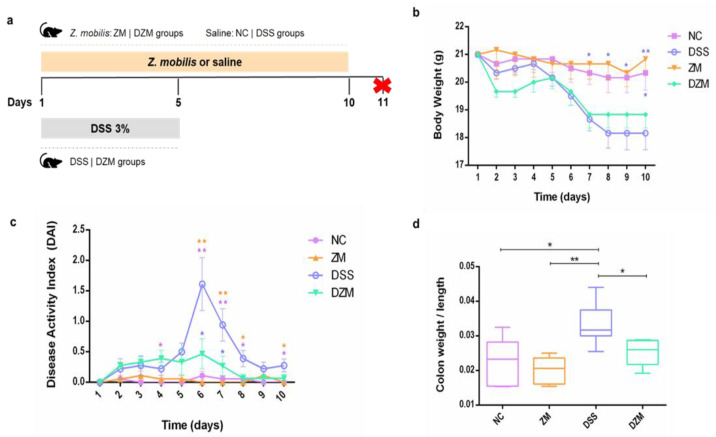
Oral administration of *Z. mobilis* and its effects on DSS-induced colitis in mice. (**a**). Animal experimental design: groups of six female C57BL/6 mice were used. Those from DSS and DZM groups ingested filtered water containing 3% DSS in the first 5 days for colitis induction. Throughout the experiment, the animals from the ZM and DZM groups were gavaged with *Z. mobilis*, while the NC and DSS groups were gavaged with saline. On day 11, all animals were euthanized (represented by the red X). (**b**). The average body weight (g) of the animals was measured during the ten days of the experiment. Data are shown as the mean ± SEM, and each asterisk color represents the group for which there was a significant statistical difference. Statistical analysis was performed using one-way ANOVA with the Tukey test, * *p* < 0.05 and ** *p* < 0.01. (**c**). DAI was calculated over the entire experiment period. Data are shown as the mean ± SEM, and each asterisk color represents the group for which there was a significant statistical difference. Statistical analysis was performed using one-way ANOVA with the Tukey test, * *p* < 0.05 and ** *p* < 0.01. (**d**). Colon weight/length ratio (cm) after euthanasia. Data are shown as the median and SD (*n* = 6). Statistical analysis was performed using the Mann–Whitney test, * *p* < 0.05 and ** *p* < 0.01.

For acute colitis induction, dextran sulfate sodium salt—DSS (MP Biomedical, Irvine, CA, USA) with a molecular weight range of 36,000–50,000 and a concentration of 3% (*w*/*v*) was added to the water of animals in the DSS and DZM groups during the first five days of the experiment, followed by the ingestion of normal water (Figure 1a). The NC and ZM groups consumed water without DSS throughout the period. Liquid consumption was monitored, and all groups consumed similar volumes of liquid, equivalent to an average of ±5 mL per mouse/day. The experiment was carried out in independent replicates.

Mouse weights were recorded throughout the experiment, and fecal samples were collected on days 5 and 10 and stored at −80 °C until analysis. Euthanasia was performed by administering a sublethal dose of ketamine (300 mg) and xylazine (30 mg) anesthetics on the final day of the experiment (day 11). Colons was collected and divided into two parts, one for RNA extraction (maintained with RNAlater Stabilization Solution—Thermo Scientific, Waltham, MA, USA) and the other for histological evaluation (maintained in 10% paraformaldehyde).

### 2.4. Disease Activity Index (DAI)

The severity of DSS-induced colitis was measured by macroscopic parameters using the disease activity index (DAI) [13]. This index stipulates values (score) for body weight loss (0, none; 1, 1–5% loss; 2, 5–10% loss; 3, 10–20% loss; and 4, >20% loss), stool consistency (0, normal; 2, loose stools and 4, diarrhea) and rectal bleeding (0, absent; 2, moderate and 4, severe). Body weight loss was defined as the difference between initial and final weight. Stool consistency and rectal bleeding were determined by daily visual assessment of the animals. Colon weight and length were evaluated after mice euthanasia.

### 2.5. Histopathological Analysis

Colon samples were collected and fixed in 10% paraformaldehyde, then embedded in paraffin; histological sections were stained with hematoxylin and eosin (H&E). The slides were scanned with Aperio CS2 equipment (Leica, Wetzlar, Germany) and viewed under 20× and 40× magnification power with Aperio ImageScope Software, v12 (Leica, Wetzlar, Germany). Blind histological analyses were performed considering morphological changes in the following parameters: inflammatory cell infiltrate in terms of severity and extent; epithelial changes, which include degrees of hyperplasia, goblet cell loss, cryptitis, crypt abscess, and erosion; mucosal architecture damage such as ulceration, presence of irregular crypts, and crypt loss. The scores were assigned as previously described by Erben et al. (2014) [14].

### 2.6. Microbiome Analysis

Three weeks before the beginning of the experiment, the mice went through a co-housing process: once a week, the animals were randomly mixed in the cages to homogenize the microbiota. Feces from days 5 and 10 of all animals were collected. To verify changes in the profile of microbial diversity in the group and not just individually, a pool of samples from each group was carried out on the established days. Total bacterial DNA from the feces pool was extracted with the GenElute™ Stool DNA Isolation Kit (Sigma-Aldrich, St. Louis, MO, USA). The V3–V4 region of the 16S ribosomal RNA gene was sequenced to generate 250 bp paired-end raw reads using the HiSeq platform (Illumina Inc., San Diego, CA, USA) by GenOne Biotechnologies (Rio de Janeiro, Brazil).

Paired-end reads were merged using FLASH (V1.2.7) [15]. Quality filtering was performed on raw tags to obtain high-quality clean tags according to QIIME (V1.7.0) [16,17]. The tags were compared with the SILVA 138 reference database using UCHIME algorithm to detect and remove chimera sequences [18,19,20]. Effective tags were finally obtained, and sequence analysis was performed by Uparse software, v11 [21]. Sequences with ≥97% similarity were assigned to the same OTUs (operational taxonomic units). To obtain the phylogenetic relationship of all OTs representative sequences, the MUSCLE software, v5 was used (V3.8.31) [22]. Species richness and evenness (alpha diversity) were measured using the Chao1 and Shannon indexes, and beta diversity was calculated with QIIME. A heatmap was plotted using SRPLOT (www.bioinformatics.com.cn/en (accessed on 1 July 2023)). The raw sequencing data have been uploaded to the NCBI Sequence Read Archive (SRA) database under BioProject number PRJNA997586.

### 2.7. RNA Isolation and Real-Time PCR Analysis

A fraction of the intestinal colon was macerated using TissueLyser LT (Qiagen, Hilden, Germany) to disrupt the tissue. The extraction of total RNA was performed using the RNeasy^®^ Protect Mini Kit (Qiagen, Hilden, Germany), according to the manufacturer’s instructions. Quantifications of RNA samples were performed using a NanoDrop One spectrophotometer (Thermo Scientific, Waltham, MA, USA). To eliminate genomic DNA from RNA samples, TURBO™ DNase (Invitrogen, Waltham, MA, USA) was used, followed by reverse transcription with the High-Capacity cDNA Reverse Transcription Kit (Applied Biosystems, Waltham, MA, USA). Assays were performed in duplicate using the LightCycler^®^ RNA Master SYBR Green I kit (Roche, Basel, Switzerland) in ABI Step One Plus Real-Time PCR System equipment (Applied Biosystems, Waltham, MA, USA). Relative mRNA expression was normalized from endogenous gene beta-2-microglobulin (*B2m*) expression, and the 2^−ΔΔCt^ method was used to calculate transcript levels. The oligonucleotides are described in Supplemental Table S1. Data are expressed as the mean ± standard error of the mean. Real-time pCR fold changes were calculated using RT2 Profiler PCR Array Data Analysis software, v5.1 (Qiagen, Hilden, Germany).

### 2.8. Statistical Analysis

The results of body weight and DAI experiments are expressed as means ± SEM. Colon weight/length ratio results are expressed as medians ± SD and real-time PCR and histological score results are expressed as means ± SD. Statistical differences were determined by one-way ANOVA with a Tukey post hoc test for body weight and DAI curves and by the Mann–Whitney test for colon weight/length ratios, histological scores, and real-time PCR charts. All statistical analyses were performed with GraphPad Prism version 6.0 (La Jolla, CA, USA) and *p*-values were considered significant <0.05.

## 3. Results

### 3.1. Oral Administration of Zymomonas mobilis Attenuates DSS-Induced Colitis In Vivo

The effects of *Z. mobilis* ingestion on DSS-induced colitis in mice were evaluated. The experiment lasted 10 days. From the first to the fifth day of the experiment, the animals in the DSS and DZM groups consumed DSS 3% in their water, while the NC and ZM groups only drank water. The animals were evaluated daily for weight, stool consistency, and degree of rectal bleeding. The animals were orally gavaged throughout the experiment: the ZM and DZM groups with 100 μL *Z. mobilis* (5 × 10^9^ CFU/mL) and the NC and DSS groups with 100 μL of saline. On the 11th day, all animals were euthanized (Figure 1a).

Body weights showed a gradual decrease in the experimental groups during the first five days of DSS treatment, with a markedly accelerated decline in the DSS-treated groups from the fifth day until the end of the experiment (Figure 1b). *Z. mobilis* (ZM) treatment did not impact body weight. The DZM group lost weight during DSS treatment, which stabilized after day 8.

The disease activity index (DAI) quantifies the macroscopic parameters of DSS-induced colitis based on weight loss, stool consistency, and rectal bleeding. The higher this index, the more intense the macroscopic symptoms and the sicker the animals are. The effect of *Z. mobilis* treatment on murine colitis was assessed using these indexes during the 10 days of the experiment. The DSS group had the highest DAI throughout the experiment and peaked on the sixth day. The peak was not observed for induced colitis mice treated with *Z. mobilis* (DZM group). After halting DSS administration, DAI scores declined until day 10, where most groups were comparable, except the DSS group (Figure 1c).

Intestinal inflammation can be assessed by measuring the size and weight of the colon [23,24,25,26]. Colon weight/length ratio reflects the intestinal inflammatory status. The ratio was significantly different between the groups that received DSS and the group that received DSS along with *Z. mobilis*—DZM (*p* < 0.05) (Figure 1d). The data shown in Figure 1b–d suggest that the presence of *Z. mobilis* in the gastrointestinal tract of mice may have ameliorated disease progression, controlling weight loss and severe symptoms in these animals.

These data also corroborate with a previous pilot experiment, in which the DZM group treated with *Z. mobilis* only in the last six days of the experiment (14 days—Supplemental Figure S1a) showed DAI and weight stability and a low colon weight/length ratio (Supplemental Figure S1b–d).

### 3.2. Zymomonas mobilis Alters Mice Colon Mucosa

The progression of DSS-induced colitis is marked by an increase in inflammatory cell infiltration into the colonic mucosa, leading to the destruction of colonocytes and raising inflammatory activity [27]. To explore whether treatment with *Z. mobilis* could alleviate colitis by regulating inflammation and decreasing mucosal damage, histological analysis of the colon with H&E staining was performed [14].

**Figure 2:**
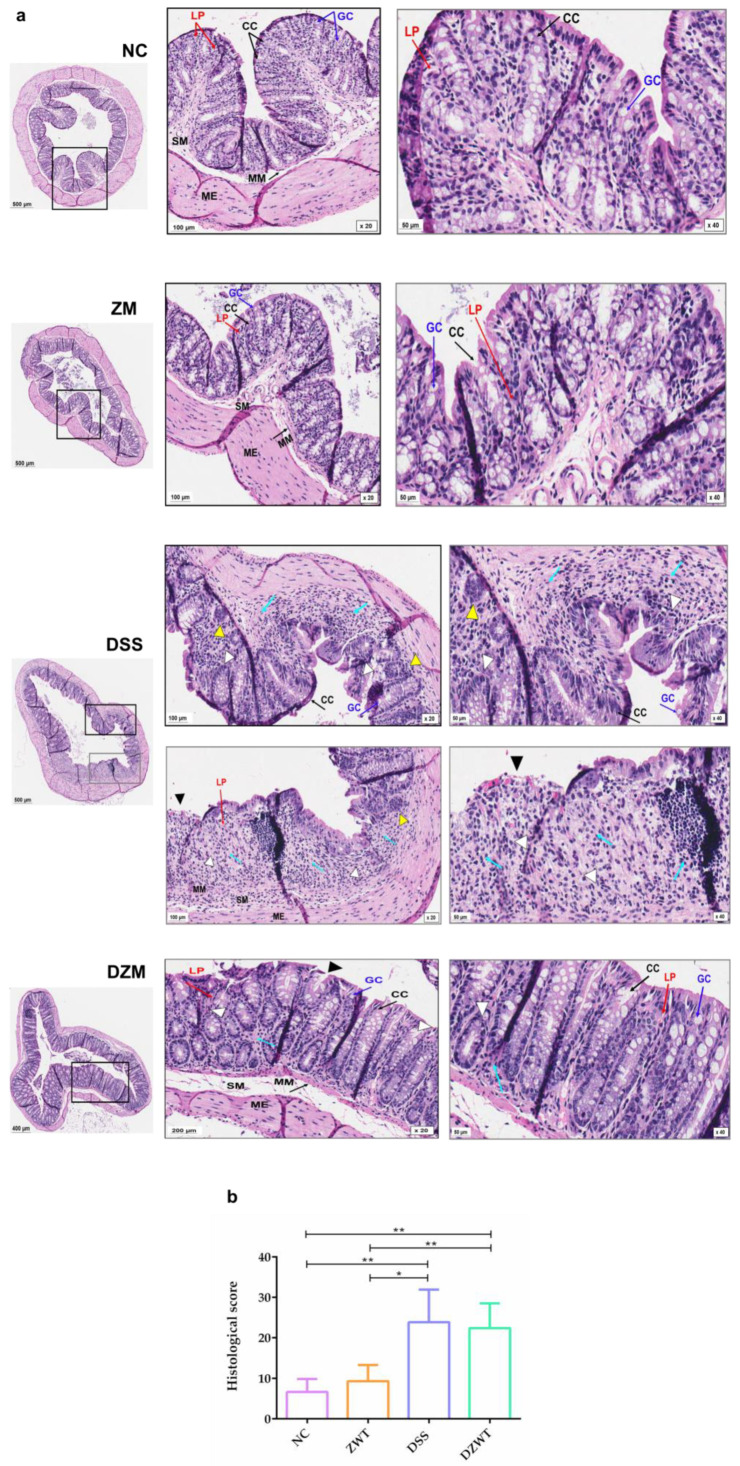
Histopathological analysis and effects of *Z. mobilis* on mouse colon mucosa. (**a**). Representative H&E staining of colon tissues in the NC, DSS, ZM, and DZM groups. LP: lamina propria; GC: goblet cell; CC: colon crypts; MM: muscularis mucosae; SM: submucosa; ME: muscularis externa; inflammatory cells increased in the lamina propria (blue arrow); crypt architectural disarray/crypt loss/irregular crypts/crypts with hyperplasia (white arrowhead); erosion (black arrowhead); crypt abscess (yellow arrowhead). Slides were analyzed under 20× (scale bar 100 µm) or 40× (scale bar 50 µm) magnification power with Aperio ImageScope Software (Leica, Wetzlar, Germany). (**b**). Histological score of colon tissues. Data are shown as means and SD (*n* = 6). Statistical analysis was performed using the Mann–Whitney test: * *p* < 0.05 and ** *p* < 0.01.

The DSS-treated group showed classic signs of induced colitis, such as damaged mucosa and inflammatory cell growth in the lamina propria (blue arrow); crypt architectural disarray, crypt loss, irregular crypts, and crypts with hyperplasia (white arrowhead); loss of surface epithelium—erosion (black arrowhead); crypt abscess (yellow arrowhead); focal ulceration and decreased goblet cells (Figure 2a).

The group that was orally gavaged with *Z. mobilis* (ZM) only was morphologically similar to the healthy group (NC). In mice that received DSS and *Z. mobilis* (DZM), histological damage in the mucosa was ameliorated, and it was possible to observe few areas of erosion in the epithelium, a decrease in inflammatory cells, regular crypts with areas of hyperplasia, and an increase in goblet cells. In addition, the group that received only DSS showed stronger inflammatory signs in an overall damaged mucosa. The histological score is shown in Figure 2b.

### 3.3. Administration of Z. mobilis Alters Gut Microbiota

The V3–V4 regions of the 16S rRNA gene were sequenced from pooled animal stool samples to evaluate the effects of *Z. mobilis* on the gut microbiota. Fecal samples were collected from six animals from the NC, DSS, ZM, and DZM groups on days 5 and 10.

**Figure 3:**
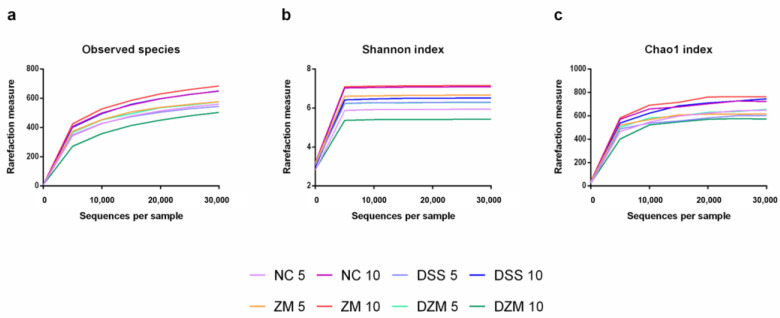
Alpha diversity analysis. (**a**). Observed species in each sample pool on days 5 and 10. (**b**). Shannon diversity index measured in each sample pool on days 5 and 10. (**c**). Chao1 richness index measured in each sample pool on days 5 and 10.

Alpha diversity is an analytic method to assess community complexity in each sample through specific metrics [28]. Part of this analysis index is the number of observed species that presented a lower rarefaction plateau in the DZM10 sample (Figure 3a).

Bacterial community diversity, assessed by the Shannon index, showed an increase in the ZM 10 and NC 10 groups, in contrast to groups DZM 10 and NC 5, which had a lower species diversity (Figure 3b). Another alpha diversity analysis index is Chao1, which evaluates the richness of bacterial communities. Based on the sequencing data, the ZM 10, NC 10, and DSS 10 groups presented increased rarefaction indexes in relation to the other groups (Figure 3c).

**Figure 4:**
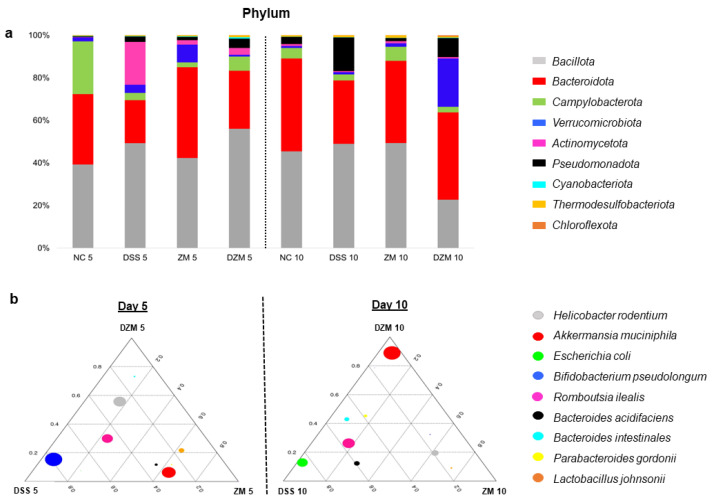
Composition of gut microbial community in mice. (**a**). Histogram of relative abundance of dominant phyla on days 5 and 10. (**b**). Ternary plots of relative abundance of dominant species on days 5 and 10 in which each corner of the triangle represents a time point (D5, NZW5, DZW5 or D10, NZW10, DZW10) and each circle size represents species abundance.

The topmost relative abundant microbial communities were identified in the composition of each sample taxa at the phylum level (Figure 4a). In a general comparative analysis, the most abundant phyla in all samples during the days analyzed were *Bacillota* and *Bacteroidota*. *Actinomycetota* was more abundant in the DSS group on day 5. On day 10, there was an increase in *Pseudomonadota* in the DSS and DZM groups, and in the latter group, there was also an increase in *Verrucomicrobiota*. The phyla *Cyanobacteriota*, *Thermodesulfobacteriota*, and *Chloroflexota* remained stable during the analyzed days.

At the species level, more intense changes occurred among the groups on days 5 and 10, as shown by ternary plots (Figure 4b). DSS treatment was associated with a dominant presence of *Bifidobacterium pseudolongum* on day 5 (DSS 5) and *Escherichia coli* on day 10 (DSS 10), as well as *Romboutsia ilealis* at both time points. In contrast, the group that received DSS plus treatment with *Z. mobilis* increased the relative abundance of the bacteria *Akkermansia muciniphila* at day 10 (DZM 10) after its increased abundance in the ZM group at day 5.

**Figure 5:**
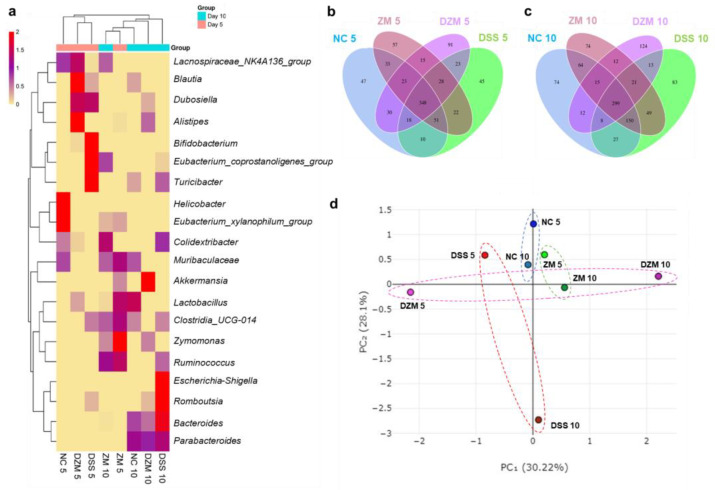
Microbiome diversity analysis. (**a**). Heatmap of topmost abundant genera of mice gut microbiota groups NC, DSS, ZM, and DZM on days 5 and 10 of analyzes. (**b**,**c**). Venn diagram of OTUs distributed among groups on day 5 and day 10, respectively. (**d**). PCA plot points represent different sample groups and their clusters.

To evaluate the relationship between the bacteria genus distribution in the microbiota of the control samples (NC and DSS) and those that received *Z. mobilis* (ZM and DZM), a heatmap analysis was used with the 20 most abundant common genera among all samples. The samples were clustered by their abundance distribution in different samples (by the Euclidean distance method) (Figure 5a). Interestingly, groups from the same day tended to cluster together. Also, of note is the abundance of *Akkermansia* in the DZM group and Escherichia-Shigella in the DSS group on day 10. The *Zymomonas* genus is not among the most abundant. However, it was added to the heatmap as it is the object of our work.

According to the results of OTU clustering analysis, a Venn diagram was generated with common and unique information for the different samples on days 5 (NC 5, DSS 5, ZM 5, and DZM 5) and 10 (NC 10, DSS 10, ZM 10, and DZM 10) (Figure 5b,c). The reduction in the number of common species (348 to 299) and the rise in unique OTUs in all groups on day 10 are remarkable. There is also an increase in shared OTUs between the NC 10 and DSS 10 groups in relation to the NC 5 and DSS 5 groups, suggesting a possible recovery of homeostasis in the DSS group.

Beta diversity was also observed, using principal component analysis (PCA). The control group measurements on days 5 and 10 appear close to each other, similarly to those for the group that received *Z. mobilis* only, both clustering in the middle of the graph. However, neither DSS group clusters, and their day 5 and day 10 sample results are spread antagonistically (Figure 5d).

### 3.4. Zymomonas mobilis Treatment Suggests Regulation of the Inflammatory Response in the Experimental Colitis Model

Genes that play a key role in mediating the immune response in inflammatory diseases were analyzed to evaluate the impact of *Z. mobilis* treatment on modulating the immune response. The expression of these genes was evaluated by real-time PCR with colonic mRNA from animals in the NC, DSS, and DZM groups at the end of the experiment.

**Figure 6:**
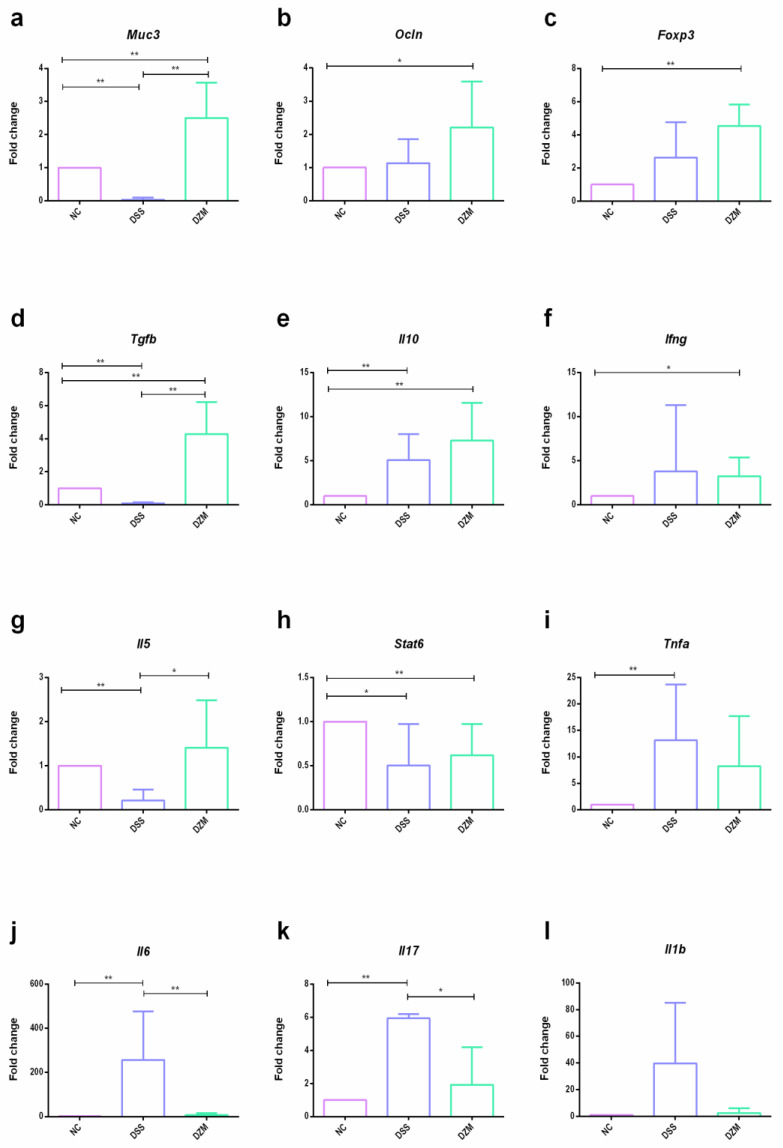
Colonic mRNA expression by real-time PCR analysis. Relative gene expression: (**a**). *Muc3*; (**b**). *Ocln*; (**c**). *Foxp3*; (**d**). *Tgfb*; (**e**). *Il10*; (**f**). *Ifng*; (**g**). *Il5*; (**h**). *Stat6*; (**i**). *Tnfa*; (**j**). *Il6*; (**k**). *Il17*; (**l**). *Il1b*. Data are shown as means and SD (*n* = 6). Statistical analysis was performed using the Mann–Whitney test: * *p* < 0.05 and ** *p* < 0.01.

Molecular markers such as Mucin 3 (Muc3) and Occludin (Ocln) are related to mucus production by goblet cells and the integrity of tight junctions in colonocytes, respectively. Their expression reflects tissue restoration. DSS treatment seems to have impaired *Muc3* expression, but treating with *Z. mobilis* activated it. Ocln expression was not affected after DSS treatment, but it was induced by *Z. mobilis* supplementation. Consistently with histopathological analyses, the expression of these markers increased in the DZM group compared to the DSS and NC group (Figure 6a,b).

DSS treatment increased inflammatory markers such as *Il6*, *Il17* and *Tnfa*, and the two formers were reversed with *Z. mobilis* treatment. Moreover, the DZM group showed a significant increase in the gene expression of transcription factor *Foxp3* and cytokines *Tgfb*, *Il10*, and *Il5,* associated with a regulatory profile, compared to NC and DSS groups (Figure 6c–g). The DSS and DZM groups also showed a significantly decreased expression of the *Stat6* transcription factor, which is related to colitis development and epithelial tight junction disruption [29] (Figure 6h). Other inflammatory markers, such as *Ifng*, *Il1b* (Figure 6f,l), *Rorc*, and *Il22* (Supplemental Figure S2), were also tested, but did not show statistically significant differences.

## 4. Discussion

Chronic inflammation of the bowel is a modern-day disease that affects a large number of individuals, principally in industrialized countries. Even though no specific treatment is available, the use of probiotics could lead to benefits in terms of disease control. For example, *E. coli* Nissle 1917 (Mutaflor, Ardeypharm) has been shown to induce remission of UC in patients and maintain it for a certain period [30,31]. This effect was also compared with the drug aminosalicylate mesalamine, one of the main treatments for UC [32,33,34]. Another relevant clinical trial used a bacterial mixture VSL#3 (VSL Pharmaceuticals, Inc.), which combines eight species of bacteria from the genera *Bifidobacterium*, *Lactobacillus*, and *Streptococcus*. This medication proved to be safe and effective in reducing the symptoms caused by UC, in addition to inducing and maintaining remission of this disease for a period in the patients tested [35,36,37]. In this context, the bacterium *Z. mobilis* appears as a possible therapeutic candidate as it presents probiotic characteristics associated with GRAS status and its cultural use [3,4,5], combined with the promising results obtained in this work.

The effects of *Z. mobilis* consumption on DSS-induced colitis in mice were evaluated. This experimental design is accepted as a model for human IBD due to the clinical and histopathological damage it generates in the intestinal mucosa of mice, similar to the damage that occurs in humans affected with ulcerative colitis [38,39]. This work assesses, in a preliminary way, the efficacy and mechanisms of action of possible probiotics for IBD treatment.

After a pilot test, we designed a 10-day protocol to test DSS administration in *Z. mobilis*-treated mice. The new protocol essentially resulted in the same observations. Animals in the DSS and DZM groups that drank water with DSS 3% during the first five days showed a gradual weight loss, which stabilized after the seventh day after DSS withdrawal. The NC and ZM groups remained stable and very similar throughout the experiment. Interestingly, the DZM group presented DAI results similar to the NC and ZM control groups, with a statistical difference between it and the DSS group, suggesting that this was possible due to *Z. mobilis*. Another evaluated parameter that suggested the use of *Z. mobilis* can alleviate the symptoms caused by DSS use is the significant reduction in colon weight/length ratio, whose increase is related to the intensity of the inflammatory process, observed in the DZM group in relation to the DSS group. These macroscopic evaluation features related to the severity of DSS-induced colitis suggest that disease progression and symptoms decreased in the presence of *Z. mobilis* in the gastrointestinal tract of mice.

The effect of DSS administration on the murine colon is the loss of intestinal epithelial cell barrier integrity. Due to these injuries, the tissue is exposed to microorganisms in the intestinal lumen, causing an acute inflammatory response [40,41]. This inflammatory process cause crypt architectural disarray and may lead to loss of crypts or irregular rearrangements such as hyperplasia; other histological changes typical of DSS-induced colitis are an increase in inflammatory cells in the lamina propria, erosion, ulceration, and decreased goblet cells [38,39].

All these typical changes were found in the DSS group colonic sections, unlike those of the NC and ZM groups, which presented healthy colons with an integral histological architecture. In the DZM group, an improvement in the colonic mucosa was observed, such as decreased inflammatory cells in the lamina propria, crypts recovering their regular appearance, and an increase in goblet cells. However, as the DSS treatment was suspended on day 5, some of these microscopic changes was found to have been reversed at the end of the experiment, which was reflected in the low statistical relevance of the histological score. Part of this rapid recovery may have been affected by sex. DSS-induced colitis is known to be biased by sex. Hormonal issues seem to make female mice less prone to develop colitis-associated inflammatory symptoms [42,43]. In the present work, we only used female mice to achieve a less variable response and to reduce type I error.

Mucins, the glycoproteins that make up mucus and are produced by goblet cells, play an important role in protecting the intestinal mucosal barrier. Mucin 3 is expressed in the colon and secreted in response to inflammatory cytokines. It is related to IBD pathophysiology, such as in ulcerative colitis [44,45]. However, we observed a reduction in the levels of *Muc3* mRNA in DSS-treated mice, even after five days without DSS consumption, indicating a persistent degradation of the mucus layer, keeping inflammation levels high. Conversely, the DZM group showed a significant increase in the expression of *Muc3* compared with the DSS group, suggesting a process of restoration of the mucosal barrier, possibly due to the ingestion of *Z. mobilis*.

The other structural protein studied, Occludin, is a component of intercellular tight junctions which promotes this barrier’s structural integrity [46,47]. Its expression is associated with the recovery of the intestinal mucosa. The group that received DSS and was treated with *Z. mobilis* (DZM) showed a significant increase in the *Ocln* gene in relation to the control group. These data corroborate the hypothesis that the presence of *Z. mobilis* could reestablish the integrity of the colonic epithelium, improving tissue recomposition and controlling the inflammation caused by DSS administration.

Ulcerative colitis is also related to intestinal microbial dysbiosis in humans, with decreased diversity and abundance of microorganisms [48,49]. DSS administration also changes the distribution pattern of microbial taxa in mice [38,41]. Samples from the same group were pooled before 16S rRNA gene sequencing and then used to estimate community-level microbiome diversity, as performed by Ray et al. (2019) and Rodríguez-Ruano et al. (2020) [50,51].

Microbiota were observed to change during the experiment, and in the end, five days after DSS withdrawal (day 10), animals from the DSS and DZM groups showed a marked change in the distribution profile of the phyla. A marked increase in *Pseudomonadota* (replacing *Actinomycetota*) occurred in the DSS group with an abundance of *E. coli* at the species level. The presence of this microorganism, often opportunistic, is present both in cases of ulcerative colitis in humans and in murine models and may worsen the severity of the disease [52,53]. This increase in *Actinomycetota* phyla in the DSS group on day 5 and *Pseudomonadota* on day 10 was also found in murine models of colitis induced by DSS [38,41].

The DZM group on day 10 showed a decrease in the *Bacillota* and an increase in the *Verrucomicrobiota* phylum, with an emphasis on the presence of the bacterium *Akkermansia muciniphila*. This bacterium plays a role in regulating intestinal barrier function and is involved in the host’s immune and metabolic responses [54]. Furthermore, *A. muciniphila* can modulate the expression of genes related to mucus production, since it uses these glycoproteins as an energy source [55]. Qu et al. (2021) reported a relationship between *A. muciniphila* and increased *Muc3* gene expression, also observed in our study in *Z. mobilis*-treated mice [56]. Moreover, this microorganism is reduced in patients with IBD, contrasting with its marked presence in healthy individuals [50,53]. The improved abundance of *A. muciniphila* after *Z. mobilis* consumption needs further clarification due to the statistical limitation of this finding, but it could represent a mechanism of action for *Z. mobilis* on experimental colitis.

In intestinal inflammation, the immune mechanism of action also occurs by activating genes that encode cytokines, chemokines, and other important factors for maintaining intestinal homeostasis [57]. Given the action of injury, infiltration of microorganisms, and inflammatory cells in the mucosa caused by DSS administration in mouse and human UC, cytokines associated with the inflammatory response, such as IL-6, TNF, and IL-17, all induced here by DSS, are important clues for understanding the progression of the disease [58].

In ulcerative colitis, IL-6, together with TNF, mediates the intestinal inflammatory response [57,59,60]. The cytokine TNF is highly upregulated in IBD patients. It is related to damage to the integrity of the intestinal mucosa, necrosis of Paneth and goblet cells, neoangiogenesis, and macrophage activation to enhance the production of inflammatory cytokines [60,61].

In our study, the relative mRNA expression of the proinflammatory cytokines *Tnfa*, *Il6*, and *Il17* was induced by DSS, but *Il6* and *Il17* were significantly decreased in the DZM group, suggesting that *Z. mobilis* alleviated the inflammatory response.

Transforming growth factor β (TGF-β) is an important cytokine in T-cell-mediated tolerance, inducing regulatory T cell (Treg) differentiation concomitant to Foxp3 expression, in addition to being involved in cell growth control, production of extracellular matrix, and intestinal mucosa regeneration [62,63]. DSS reduces the expression of *Tgfb* while *Z. mobilis* coadministration induces it, compared to non-treated mice. This, combined with a decrease in *Il17*, may result in the induction of regulatory cells. Another cytokine related to regulatory T cells is IL-10, which can reduce the expression of inflammatory cytokines such as TNF and IL-6. The inactivation of IL-10 causes the condition of chronic colitis, with an increase in several inflammatory cytokines [57]. In our study, both DSS and *Z. mobilis* induced *Il10*, but *Z. mobilis* administration seemed to potentiate the regulatory phenotype by efficiently inducing a broader range of immunomodulatory genes, including *Tgfb*, *Il5* and *Foxp3*.

## 5. Conclusions

The presented data suggest the ability of *Z. mobilis* to reduce disease progression in the intestinal colon in a murine model of DSS-induced colitis by reestablishing the integrity of the colonic epithelium, beneficially altering the microbiota, and significantly relieving inflammation caused by DSS administration. Despite the limitations of this work, this is a pioneering study using *Z. mobilis* in a murine model of DSS-induced colitis. It paves the way for new studies to be carried out using this innovative microorganism to ameliorate inflammatory intestinal diseases.

## Data Availability

Data from the 16S rDNA analysis are available from the NCBI Sequence Read Archive (SRA) database under BioProject number PRJNA997586. All data are available from the corresponding author on request.

## Supplementary Materials

The following supporting information can be downloaded at: https://www.mdpi.com/article/10.3390/microorganisms11112793/s1, Figure S1: Pilot experimental analysis of oral administration of *Z. mobilis* and its effects on DSS-induced colitis in mice. Figure S2. Colonic mRNA expression by real-time PCR analysis. Table S1. Primers used for real-time qPCR analysis.
