# Supplementary material for: Therapeutic Effects of Zymomonas mobilis on Experimental DSS-Induced Colitis Mouse Model
Source: Microorganisms. 2023 Nov 17;11(11):2793. doi: 10.3390/microorganisms11112793 (PMC10672878; doi:10.3390/microorganisms11112793)
Supplement: Supplementary file 1 [file microorganisms-11-02793-s001.zip › microorganisms-2693782-supplementary.pdf]

Microorganisms

Therapeutic effects of *Zymomonas mobilis* in experimental DSS-induced colitis mouse model

Manuela Maragno do Almo [1,2], Isabel Garcia Sousa [1], Vitor Guimarães Olinto [1,3], Sylvia Barbosa Pinhate [1,3], José Luiz de Paula Rôlo Jivago,[4] Davi Emanuel Ribeiro de Sousa [5], Márcio Botelho de Castro [5], Marciano Régis Rubini [6], Andrea Queiroz Maranhão [1,7] and Marcelo de Macedo Brígido [1,7] *

* Correspondence: brigido@unb.br

[1] University of Brasilia, Institute of Biological Sciences, Department of Cell Biology, Brasilia, Brazil

[2] University of Brasilia, Medicine Faculty, Molecular Pathology Graduation Program, Brasilia, Brazil

[3] University of Brasilia, Institute of Biological Sciences, Molecular Biology Graduation Program, Brasilia, Brazil

[4] University of Brasilia, Institute of Biological Sciences, Brasilia, Brazil

[5] Veterinary Pathology Laboratory, Campus Darcy Ribeiro, University of Brasília, Brasília, Brazil.

[6] Embrapa Agroenergy, Laboratory of Genetics and Molecular Biology, Brasilia, Brazil

[7] Institute for Immunology Investigation, National Institute of Science and Technology (iii-INCT), Brazil

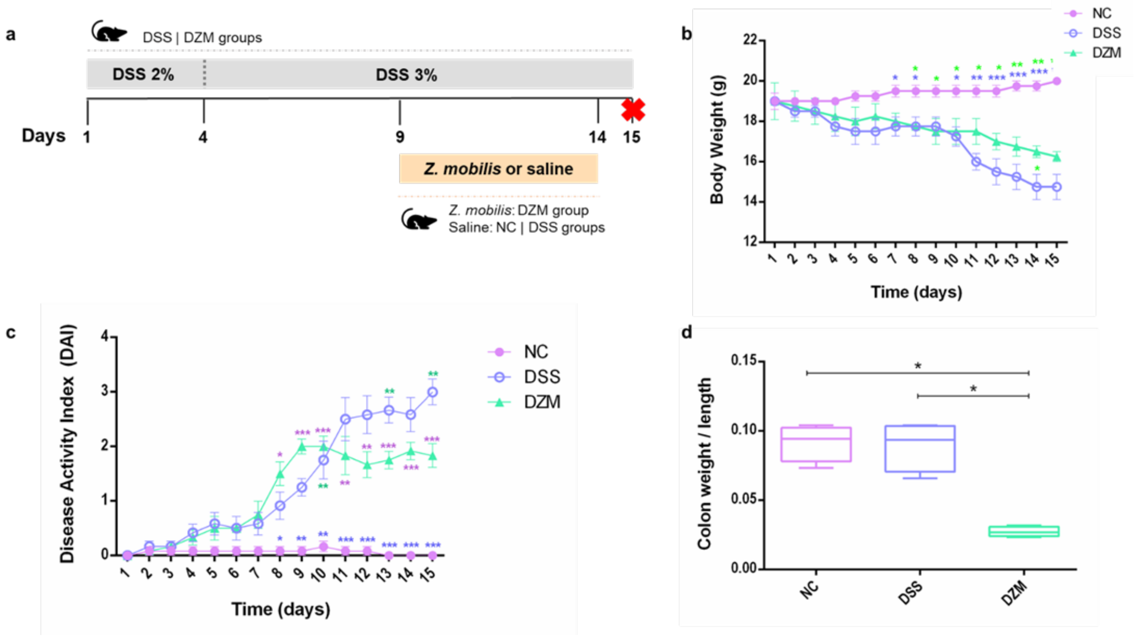

Figure S1. Pilot experimental analysis of oral administration of *Z. mobilis* and its effects in DSS induced colitis in mice. **a**. Animal experimental design: groups of four female C57BL/6 mice were used. Those from DSS and DZM groups ingested filtered water containing 2% DSS in the first 4 days for colitis induction, followed by 3% until the end of experiment. Throughout the experiment, the animals from DZM group were gavaged with *Z. mobilis*, while the NC and DSS groups, were gavaged with saline. On day 15 all animals were euthanized (represented by the red X). **b**. Average weight (g) of the animals was measured

during the fifteen days of experiment. Data are shown as the mean ± SEM and each asterisk color represents the group for which there was a significant statistical difference. Statistical analysis was performed using one-way ANOVA with Tukey test, * $p < 0.05$ and ** $p < 0.01$. **c**. Disease Activity Index (DAI) calculated over the entire period of the experiment. Data are shown as the mean ± SEM, and each asterisk color represents the group for which there was a significant statistical difference. Statistical analysis was performed using one-way ANOVA with the Tukey test, * $p < 0.05$ and ** $p < 0.01$. **d**. Colon weight/length ratio (cm) after euthanasia. Data are shown as the median and *SD*. Statistical analysis was performed using Mann-Whitney test, * $p < 0.05$ and ** $p < 0.01$.

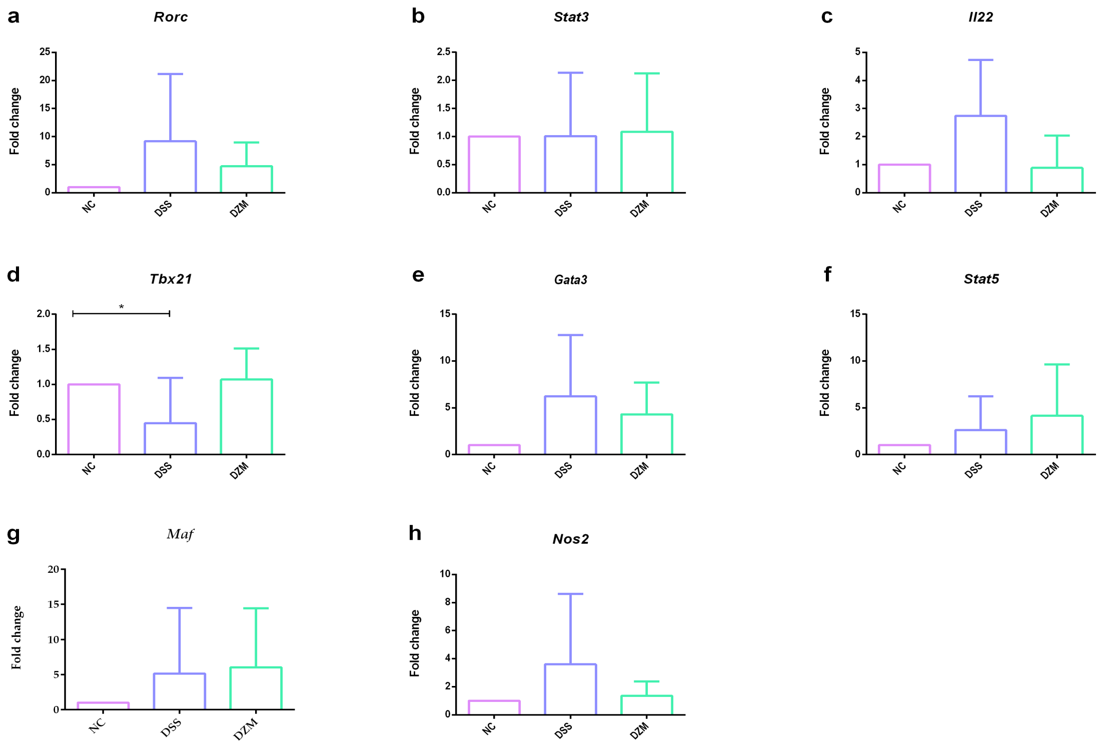

Figure S2. Colonic mRNA expression by real time PCR analysis. Relative gene expression: **a**. *Rorc*; **b**. *Stat3*; **c**. *Il22*; **d**. *Tbx21*; **e**. *Gata3*; **f**. *Stat5*; **g**. *Maf*; **h**. *Nos2*. Data are shown as the mean and SD.

Table S1. Primers used for real time qPCR analysis.

Target	Sequence
B2m	F: TGCTATCCAGAAAACCCCTCA<br>R:TTTCAATGTGAGGCGGGTGG
Foxp3	F: ACTCGCATGTTCGCCTACTT<br>R: AGGGATTGGAGCACTTGTTG
Tgfb	F: CTGACGTCACTGGAGTTGTACGG<br>R: GGTTCATGTCATGGATGGTGC
Il10	F: GGTTGCCAAGCCTTATCGGA<br>R: GAGAAATCGATGACAGCGCC
Ifnγ	F: AAGTTTGAGGTCAACAACCCAC<br>R: AATCTCTTCCCCACCCCGAA
Il5	F: F: CTCTGTTGACAAGCAATGAGACG<br>R: TCTTCAGTATGTCTAGCCCCTG
Stat6	F: GGATTCAGATTTGGAAGCGGC<br>R: AATTTCCACCAGGCTTTCACAC
Tnfα	F: GTAGCCCACGTCGTAGCAAA<br>R: ACAAGGTACAACCCATCGGC
Il6	F: GCCTTCTTGGGACTGATGCT<br>R: TGCCATTGCACAACTCTTTTCT
Il17	F: TTTAACTCCCTTGGCGCAAAA<br>R: CTTTCCCTCCGCATTGACAC
Rorc	F: TGCAAGACTCATCGACAAGG<br>R: AGGGGATTCAACATCAGTGC
Il1β	F: TGCCACCTTTTGACAGTGATG<br>R: TGTGCTGCTGCGAGATTTGA
Il22	F: GCTCAGCTCCTGTCACATCA<br>R: CAGTTCCCCAATCGCCTTGA
Stat3	F: CTGCCCCGTACCTGAAGAC<br>R: AACGTGAGCGACTCAAACTG
Gata3	F: TTTACCCTCCGGCTTCATCCTCCT<br>R: TGCACCTGATACTTGAGGCACTCT
Stat5	F: CACTCCTGTACTTGGTTCGTCA<br>R: CCAGGTCAAACTCGCCATCT
Tbx21	F: TCAGGACTAGGCGAAGGAGA<br>R: GCCTTCGGTTTCCTTATCAA
Nos2	F: GGTGAAGGGACTGAGCTGTT<br>R: ACGTTCTCCGTTCTCTTGCAG
Maf	F: GGATGGCTTCAGAACTGGCA<br>R: AACATATTCCATGGCCAGGG
Muc3	F: CGTGGTCAACTGCGAGAATGG<br>R: CGGCTCTATCTCTACGCTCTC
Ocln	F: ATGACATGTATGGCGGAGAG<br>R: ATAGCCTCTGTCCCAAGCAA
